# Design and implementation of a module for teaching and research on SCRs for power electronics

**DOI:** 10.1016/j.ohx.2025.e00640

**Published:** 2025-03-22

**Authors:** Jhon Bayona, Nancy Gélvez, Helbert Espitia

**Affiliations:** Facultad de Ingeniería, Universidad Distrital Francisco José de Caldas, Colombia

**Keywords:** Power electronics, SCRs, Teaching, Research, Converters

## Abstract

The conversion of energy is a fundamental aspect for adequate use of energy resources; thus, counting on appropriate devices and methodologies in education and research is essential. This document presents an experimental module for teaching and research on silicon controlled rectifiers (SCRs) in power systems, where different experiments designed to cover a diverse range of complexities are also proposed. In low complexity, the experiments focus on implementing phase control rectifiers and three-phase AC–AC voltage controllers, with particular attention to passive loads. On the other hand, in the field of high complexity, the experiments delve into the application of full-wave phase control three-phase rectifiers in direct current transmission systems, as well as the use of AC–AC voltage controllers in static reactive power compensators. The results show that the desired behavior is achieved according to the theory for the different experiments proposed.

## Graphical abstract

**Figure:**
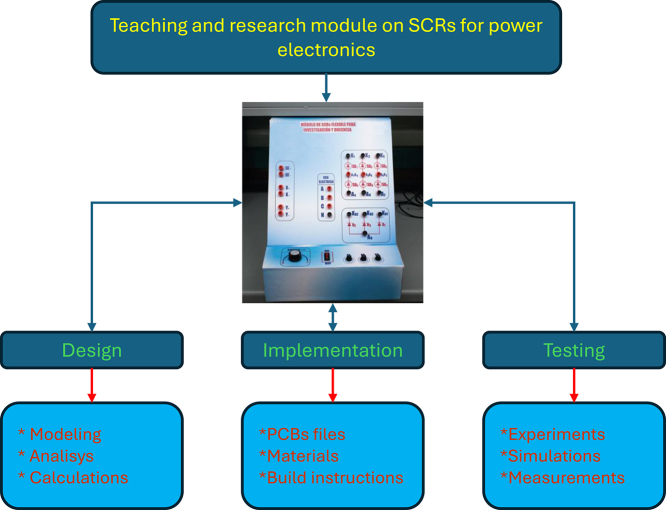

## Specifications table

**Table:** 

Hardware name	*SCRs didactic module for power electronics*

Subject area	•*Electrical engineering* •*Educational and research tools*

Hardware type	•*Electrical engineering and computer science* •*Power electronics*

Closest commercial analog	•*LEYBOLD ^Ⓡ^ Uncontrolled/controlled rectifier circuits* •*DE LORENZO - ELECTRONIC DEVICES Training Systems*

Open source license	*CC-BY-NC 4.0*
Cost of hardware	*$ 300 USD.*
Source file repository	https://doi.org/10.17632/vfsj3zhsgf.2

## 1 Hardware in context

In a global context where the rational use of energy is an issue of relevant magnitude, having the appropriate methodologies and devices to carry out the different experiments in education and research is a fundamental aspect of energy conversion; thus, this document addresses energy conversion circuits employing SCRs as a switching element, which was developed by *General Electric* in 1957. Even though considered an old semiconductor in the area of power electronics, it stands out as the currently most robust semiconductor. The SCR can handle the highest amount of power compared to other semiconductors such as BJTs, MOSFETs, and IGBTs, among others [1]. SCRs are available on the market with voltage and current values that reach thousands of volts and amps. Meanwhile, direct current transmission lines employ series-connected line-triggered SCRs. In this way, SCRs are employed in inverters and rectifiers that interconnect AC systems and DC transmission lines capable of carrying among 1 kA and 1 MV [2]. For this reason, students of first course on electrical and electronic engineering must know the SCR and its various applications in circuits that operate with line voltages of 50–60 Hz [3], these circuits are phase control rectifiers and AC–AC voltage controllers. Such rectifiers are commonly used for various applications including controlled power supplies, electrochemical processes, traction equipment, and motor drivers. Conversely, standard applications in AC–AC voltage controllers include lighting and heating control, dual feeders, dynamic voltage reclosers, static compensators, online transformer winding switching, soft starting, and speed control of motor drives for pumps and fans, among others [1], [3].

However, most universities lack of didactic modules for circuit implementation in the laboratory; as a result, students are forced to build them by themselves. This situation causes problems such as high costs and extensive construction times, commuting inconveniences, and a shortage of SCRs-related lab opportunities. On the other hand, SCRs modules are available on the market; however, many of these are expensive and need more control systems to trigger the SCRs, which limits the possibility of carrying out more complex experiments.

### 1.1 Related hardware works

Regarding development of issues related to experimental module works in education and investigation on power-electronics, Ref. [4] introduces an execution of a power-electronic remote-laboratory (ELEPOT-rLab); such experiments are capable of accomplishing actual power-electronic converter on the Internet. Remotely, users are able to control the testing and obtain graphical results of the measurements instantly.

In power electronics, a proposal and experimental project carried out by groups of students, and a teaching and problem-based learning experience is presented in [5] for using electronic equipment in consumer and industrial applications to accompany professional training progressively.

Some teaching-related issues on power electronics in higher education training courses are displayed in [6] presenting a modular methodology for teaching three-phase thyristor rectifiers. A MATLAB equation solver simulator was developed regarding the detailed physical operation of three-phase thyristor rectifiers. The developed simulator considers aspects like control parameter variations, the effects generated on the supply network, continuity and discontinuity, and commutation.

An experimental platform for teaching AC–DC converters in the subject of power electronics of the Federal University of Uberlândia (FUU), Brazil was proposed in [7]. The platform consists of either controlled or uncontrolled three-phase rectifier. The integrated circuit TCA785 is used to control the operation of the SCRs.

A concept of Education 4.0 is introduced in [8] including experiments using power electronics in real time; clearly, such concept requires current methodological education to be promoted to Education 4.0; thus, the authors of [8] work toward the association of new experiments in the laboratory, including real-time oscilloscope (remote/call) unit, augmented reality unit, and Internet of things (IoT) base unit.

Besides, [9] introduces EZ-TBOARD, which is an experimentation system that quickens the learning process to basic applicability and operation of semiconductor transistors. In the training board, throughout a reconfigurable and compact design, students obtain the necessary help to configure amplification and switching schemes, and the major different biasing in every kind of three terminal transistors aiming to dedicate all the attention only on those key aspects related to device and application circuit theory, which promotes student’s higher analysis capacity to reconfigure and design a circuit when a specific action is required.

Finally in the context of education, a flexible didactic platform for thyristor-based circuits is displayed in [10]. The proposed module allows the construction of various topologies including thyristor bridge rectifiers and solid-state relays, among others. The operation of a controlled rectifier is taken as an example of teaching applications to evidence their functional and didactic use.

Regarding to SCRs models based on state-space averaging techniques, authors in [11] apply the modeling method employing the concept of duty-ratio constraint to represent the rectifier’s electrical behavior in continuous and discontinuous operating modes. From a computer simulation with the average and the switching models the results are presented for both conditions, namely, steady-state and transient state.

### 1.2 Focus and document organization

This work proposes an experimental module for teaching and research of SCRs in power systems. The approach involves a series of experiments designed to encompass diverse complexities. In low complexity, the experiments focus on implementing phase control rectifiers and three-phase AC–AC voltage controllers emphasizing passive loads. On the other hand, highly complex experiments delve into the use of full-wave phase control three-phase rectifiers within direct current transmission systems and the application of AC–AC voltage controllers in static compensating systems of reactive power. This variety of experiments provide a comprehensive understanding of SCRs, from their fundamental applications to advanced uses in more complex power systems. This work seeks to generate a reference framework for developing experiments with SCRs in the field of open-source teaching and research, allowing access to the resources used to implement circuits and programming software in the DSP.

In the paper the hardware description occurs in Section 2, the operation of the Half Bridge SCR Gate Driver (HBSGD) circuit is presented in Section 2.1. The design of the HBSGD circuit is detailed in Section 2.2, followed by the description of the operation of the Zero Crossing Detector Led Driver (ZCDLD) circuit in Section 2.3, and its respective design in Section 2.4. Section 3 displays the design files summary, the bill of materials summary in Section 4, and the build instructions in Section 5. Meanwhile, Section 6 shows the operation instructions, and Section 7 the validation and characterization allowing to observe the performance of the proposed experiments. Finally, the discussion and conclusions regarding the results are presented in Sections 8, and 9.

## 2 Hardware description

This section presents the detailed analysis, design, and implementation of the hardware, starting with the operation analysis and design of the Half Bridge SCR Gate Driver circuit. It is followed by a description of the operation of the Zero Crossing Detector Led Driver and its design.

### 2.1 HBSGD circuit operation

**Fig. 1:**
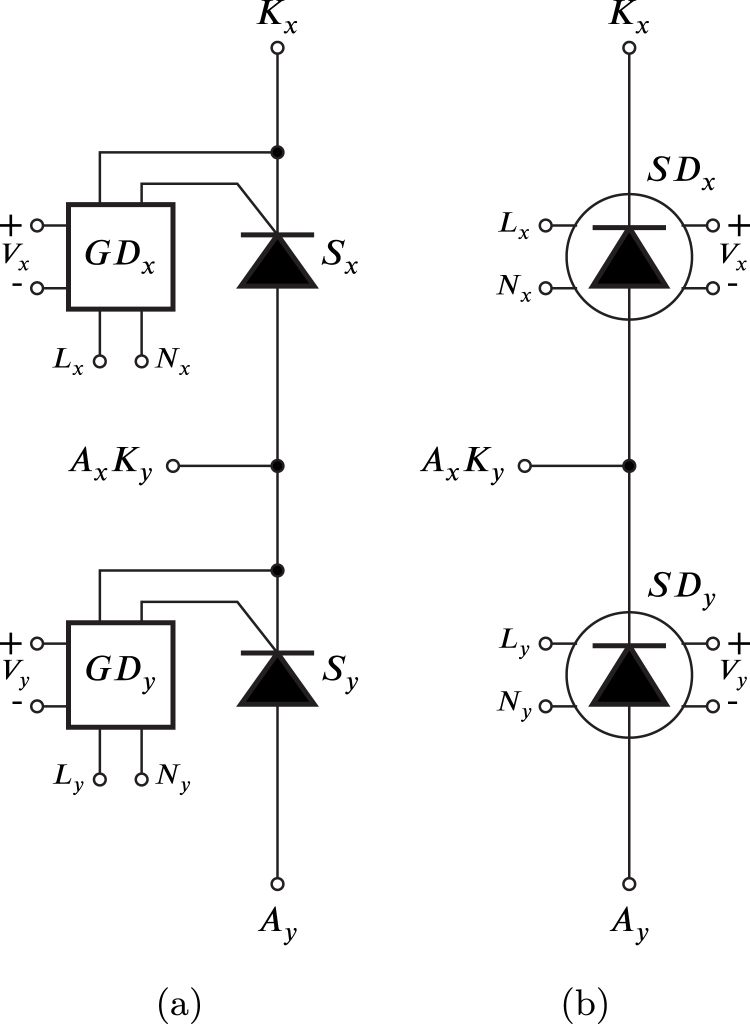
HBSGD circuit with: (a) each SCR and the gate driver, (b) simplified symbol of each SCR and its gate driver.

The proposed HBSGD circuit is depicted in Fig. 1(a); hereafter, its representation is the simplified symbol version of the SCR and its GD, as illustrated in Fig. 1(b). The junction of the $SD_{x}$ anode with the $SD_{y}$ cathode forms the $A_{x}K_{y}$ terminal, the $SD_{x}$ cathode is designated as the $K_{x}$ terminal, and the $SD_{y}$ anode is referred to as the $A_{y}$ terminal.

This form of connection generates a highly versatile half-bridge of SCRs since it allows the synthesis of a variety of circuits with SCRs that can be classified into two groups, namely, phase control rectifiers and AC–AC voltage controllers, which can be six-phase, three-phase, two-phase and single-phase, depending on the number of HBSGDs used.

**Fig. 2:**
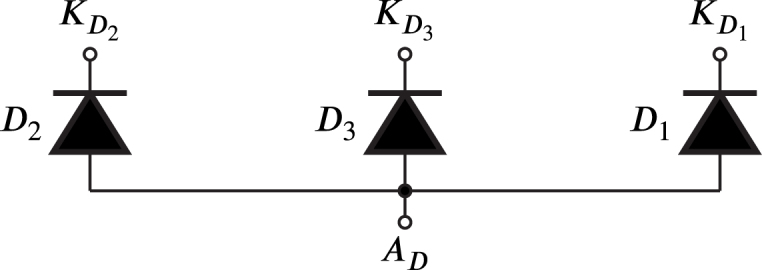
Power diodes used in the module.

In this article, a flexible SCR module is proposed for research and teaching with three HBSGDs. Full-wave and half-wave phase control rectifiers can be configured using the power diodes in Fig. 2 and AC–AC voltage controllers, these rectifiers and controllers can be, at most, three-phase.

The standard circuit for turning on the SCR, most commonly used in phase control rectifiers and AC–AC voltage controllers is a voltage source controlled in series with a resistor; however, the current produced by this circuit that flows through the gate presents variations as the gate and the cathode form a P-N junction that is highly sensitive to temperature changes, consequently, in the HBSGD the gates of the SCRs are driven using a controlled current source to avoid this inconvenience.

**Fig. 3:**
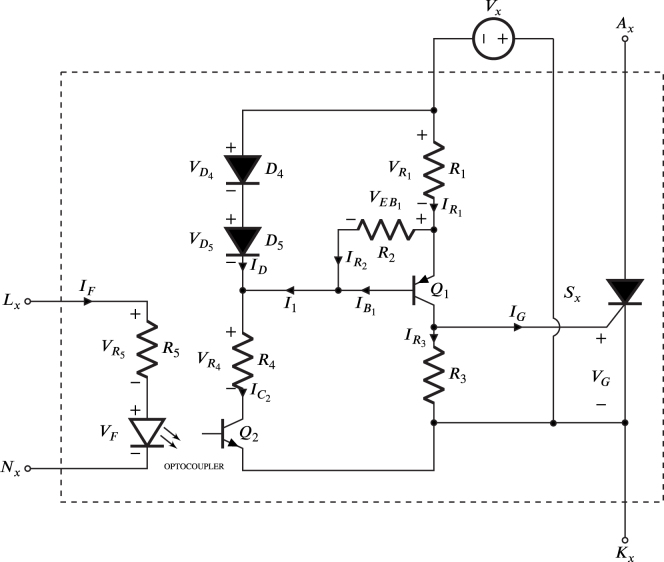
Internal structure of $SD_{x}$.

It is worth noting that $SD_{x}$ and $SD_{y}$ are identical circuits, therefore, $S_{x}$ and $GD_{x}$ are similar to $S_{y}$ and $GD_{y}$ respectively. Fig. 3 shows the $SD_{x}$ circuit, which comprises various components. $S_{x}$ is the power semiconductor SCR; $Q_{1}$, $D_{4}$, $D_{5}$, $R_{1}$, $R_{2}$, $R_{3}$ and $R_{4}$ constitutes a current-controlled source that generates the required and sufficient gate current value to transition the SCR from the off-state to the on-state. The optocoupler transistor $Q_{2}$ controls the turning on and off of the current source through the optocoupler LED. The LED current flows through $R_{5}$ and is governed by the ZCDLD circuit.

### 2.2 HBSGD circuit design

The selection of $S_{x}$ depends on the average anode forward current ($I_{A}$), when in “on state”, and the voltage stress in “off state”, which is directly proportional to the peak line voltage ($V_{L}$). It is assumed that $S_{x}$ operates in the circuit HBSGD with values for $I_{A}$ of 1 A and $V_{L}$ corresponding to 294 V. On the other hand, the considerations include the maximum average anode direct current ($I_{T}$), the maximum forward anode-to-cathode voltage ($V_{DRM}$), and the maximum reverse anode-to-cathode voltage ($V_{RRM}$). Nevertheless, either of the two voltages is taken into account, as datasheets specify equal maximum values for both forward and reverse anode-to-cathode voltages.

The maximum junction and case temperature allowed for $S_{x}$ is reached when $I_{A}$ is equal to $I_{T}$, for this reason, it is recommended to select $S_{x}$ with a $I_{T}$ that meets the condition:

(1) $$I_{T}\geq 5I_{A}\;,$$

the voltage $V_{DRM}$ is chosen high enough so that if $V_{L}$ rises, it allows a suitable margin of safety, therefore, $V_{DRM}$ is selected 30% above the peak line voltage, namely:

(2) $$V_{DRM}\geq 1.3\;V_{L}\;,$$

hence, the device SCR TYN412 is chosen for $S_{x}$, since it meets these conditions; likewise, the power diode 6A4 is selected for $D_{1}$, $D_{2}$ and $D_{3}$ having a breakdown voltage and average anode direct current similar to the chosen SCR.

The current pulse injected into the gate of $S_{x}$ must be sufficiently high to ensure a secure turn-on. Additionally, it should have a duration that suffices to prevent undesired turning off right after turning it on [1]. In order to determine the required gate current pulse magnitude to ensure the activation of $S_{x}$, it is necessary to guarantee that the gate voltage ($V_{G}$) and gate current ($I_{G}$) exceeds the gate threshold voltage ($V_{GT}$) and threshold current ($I_{GT}$), respectively. The threshold voltage and current values can be found in the datasheet for $S_{x}$. In this way, the current pulse $I_{G}$ injected into the gate must meet the condition:

(3) $$I_{G}>I_{GT}\;,$$

where $I_{GT}$ is the gate threshold current $S_{x}$ equals to 15 mA, then, if condition (3) is met, the condition:

(4) $$V_{G}>V_{GT}\;,$$

is also fulfilled, where $V_{G}$ and $V_{GT}$ are the operating and gate threshold voltages of $S_{x}$ respectively. In this case $V_{GT}$ is equal to 0.7 V. In order to avoid damage or destroy $S_{x}$, the gate operating power ($P_{G}$) must not exceed the maximum gate operating power ($P_{G\left(AV\right)}$) also listed in the datasheet of $S_{x}$, therefore, the gate operating power ($P_{G}$) must meet the condition:

(5) $$P_{G}<P_{G\left(AV\right)}\;,$$

where $P_{G\left(AV\right)}$ is the maximum gate operating power, which is equal to 1 W. Consequently, $I_{G}$ equal to 20 mA is chosen to fulfill the condition (3) having a $V_{G}$ of 0.8 V, which is obtained experimentally in the laboratory and meets the condition (4). The values of $I_{G}$ and $V_{G}$ produce a $P_{G}$ equal to 16 mW that meets the condition (5).

To continue the design process, it was considered a 5 V for supply voltage ($V_{x}$) for the circuit $SD_{x}$, typically found in phone charges widely used and low cost. The bipolar transistor 2N2907 is selected for $Q_{1}$, the diode 1N4001 for $D_{4}$ to $D_{5}$, likewise, the optocoupler PC817. These are low-cost and easy to purchase devices at electronic component stores and distributors. Regarding Fig. 3, the expression that defines $V_{R_{1}}$ is given by:

(6) $$V_{R_{1}}=V_{D_{4}}+V_{D_{5}}-V_{EB_{1}}\;,$$

since $D_{4}$, $D_{5}$, and the base-emitter of $Q_{1}$ are P-N junctions, it is assumed that they have the same forward voltage, namely:

(7) $$V_{D_{4}}=V_{D_{5}}=V_{EB_{1}}=V_{D}\;,$$

substituting (7) in (6) it is obtained:

(8) $$V_{R_{1}}=V_{D}$$

The base-to-collector leakage current at $Q_{1}$ can cause a gate current greater than zero when $Q_{2}$ is off, which is why the resistor $R_{2}$ is connected between the emitter and the base of $Q_{1}$ to guarantee a voltage $V_{EB_{1}}$ to zero forcing $Q_{1}$ to turn off. On the other hand, the resistance $R_{3}$, connected between the gate and the cathode, leads to a decrease in the possibility of turn-on due to voltage derivative, a reduction in turn-off time, and an increase in holding and latching currents in $S_{x}$. Regarding Fig. 3, the current $I_{C_{1}}$ is given by:

(9) $$I_{C_{1}}=I_{G}+I_{R_{3}}\;,$$

likewise, the current flowing through $R_{1}$, corresponds to:

(10) $$I_{R_{1}}=I_{R_{2}}+\frac{h_{FE_{1}}+1}{h_{FE_{1}}}I_{C_{1}}\;,$$

where $h_{FE_{1}}$, and $I_{C_{1}}$ are the minimum forward current gain and the collector current in $Q_{1}$ respectively. To ensure that the currents passing through $R_{2}$ and $R_{3}$ are less than 10% of $I_{G}$ are selected $I_{R_{2}}=$ 0.7 mA and $I_{R_{3}}=$ 0.25 mA satisfying this criterion. The value of $V_{D}$ is found around 0.7 V, regarding that $Q_{1}$ (Fig. 3) for an operation in active region (with a collector current of 20 mA) the approximate value of $h_{FE_{1}}$ given by the manufacturer is 120; however, the worst scenario was considered for the design, which occurs when $V_{x}$ reduces until a value where $Q_{1}$ operates on the border between the saturation and active regions. In the laboratory, it is experimentally determined that $h_{FE_{1}}$ is equal to 40 for a range of $I_{C_{1}}$ between 20 mA and 25 mA. Substituting these values into Eq. (9), and (10) the current $I_{R_{1}}$ is obtained. Meanwhile, considering Fig. 3, and Eq. (8), the current that flows through $R_{1}$ is given by:

(11) $$I_{R_{1}}=\frac{V_{D}}{R_{1}}=\text{21.65}\;\text{mA}\;,$$

in this way, it can be stated that $I_{R_{1}}$ is independent of $V_{x}$, and since $I_{G}$ is proportional to $I_{R_{1}}$, then, $I_{G}$ is a constant current source that solely depends on the voltage $V_{D}$ and the resistance $R_{1}$, which can be determined by solving for $R_{1}$ in (11) having:

(12) $$R_{1}=\frac{V_{D}}{I_{R_{1}}}=\text{32.63}\;\text{Ω}\;,$$

meanwhile, the resistances $R_{2}$, and $R_{3}$, are given by:

(13) $$R_{2}=\frac{V_{D}}{I_{R_{2}}}=\text{1}\;\text{kΩ}\;,$$

(14) $$R_{3}=\frac{V_{G}}{I_{R_{3}}}=\text{3.2}\;\text{kΩ}\;,$$

the average power dissipated by $Q_{1}$ is given by

(15) $$P_{Q_{1}}=\left(V_{x}-V_{R_{1}}-V_{G}\right)I_{C_{1}}=\text{70.8}\;\text{mW}\;,$$

since the maximum power that the bipolar transistor 2N2907 (employed for $Q_{1}$) can dissipate is 625 mW, this device can operate with the power calculated in (15) without damage. Regarding Fig. 3, the current $I_{1}$ is gyven by:

(16) $$I_{1}=\frac{I_{G}+I_{R_{3}}}{h_{FE_{1}}}+I_{R_{2}}=\text{1.2}\;\text{mA}\;,$$

aiming at maintaining $D_{4}$ and $D_{5}$ in the full conduction region, in which the forward voltage of the two diodes is between 0.6 V and 0.8 V according to datasheet, the following criterion is established for the selection of the current that flows through the two diodes:

(17) $$\text{1}\;\text{mA}<I_{D}<\text{10}\;\text{mA}\;,$$

in this way, it is selected $I_{D}$ equal to 2.2 mA fulfilling the criteria established in (17); therefore, the current flowing through $R_{4}$ is equal to 3.4 mA. On the other hand, the maximum collector-to-emitter voltage $V_{CE_{2}}$ when $Q_{2}$ is in saturation, according to the PC817 datasheet, is equal to 0.2 V. Therefore, regarding Fig. 3 the resistance $R_{4}$ is given by:

(18) $$R_{4}=\frac{V_{x}-2V_{D}-V_{CE_{2}}}{I_{C_{2}}}=\text{1}\;\text{kΩ}\;,$$

According to the PC817 datasheet, the minimum current transfer rate and maximum forward voltage $V_{F}$ of the emitter diode are equal to 0.5 mA and 1.4 V, respectively. Due to this transfer rate, the emitter diode current $I_{F}$ is equal to 6.8 mA. Reviewing Fig. 3, $L_{x}$ is a square signal with a frequency of 60 Hz and a variation range between 0 and $V_{cc}$, which produces the current $I_{F}$ when it is $V_{cc}$. This current flows through $R_{5}$ causing the saturation of $Q_{2}$. Since $L_{x}$ is a signal produced by LAUNCHXL-F28069M development board that operates with 3.3 V, then $V_{cc}$ is equal to this value, in this way, the resistance $R_{5}$ is given by:

(19) $$R_{5}=\frac{V_{cc}-V_{F}}{I_{F}}=\text{279}\;\text{Ω}$$

According to above, the current consumed by the $SD_{x}$ circuit of $V_{x}$ is the sum of $I_{R_{1}}$ and $I_{D}$, which is equal to 23.85 mA, therefore, it is recommended to select a mobile phone battery charger capable of supplying current 30% above of this value. The resistors $R_{1}$-$R_{5}$ are chosen to handle a power of 0.25 W and they are close to commercial values easy to obtain in electronic component stores and distributors. The devices $D_{4}$, $D_{5}$, $Q_{1}$ and the optocoupler PC817 operate with voltages and currents within the ranges specified by their respective datasheets.

### 2.3 ZCDLD circuit operation

**Fig. 4:**
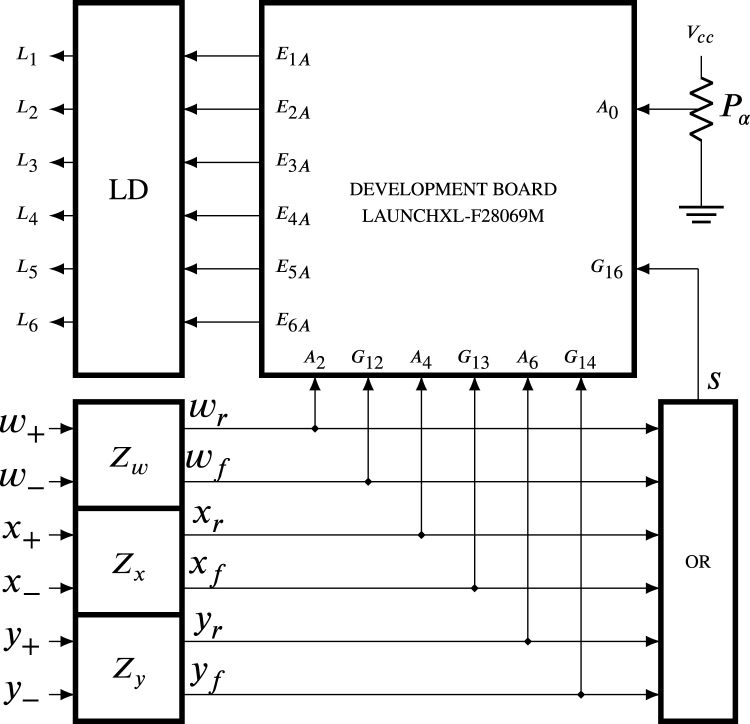
ZCDLD circuit block diagram.

The function of the circuit ZCDLD displayed in Fig. 4 is to manage the on and off of the current sources of the three HBSGD circuits to switch the SCRs from off to on state. For this, the development board LAUNCHXL-F28069M generates six signals $E_{1A},\ldots ,E_{6A}$ synchronized with the rising and falling zero crossings of the sinusoidal signals processed by the blocks $Z_{w}$, $Z_{x}$ and $Z_{y}$; hence, these blocks produce pulse signals as inputs to the development board to determine when the zero crossings occur.

**Fig. 5:**
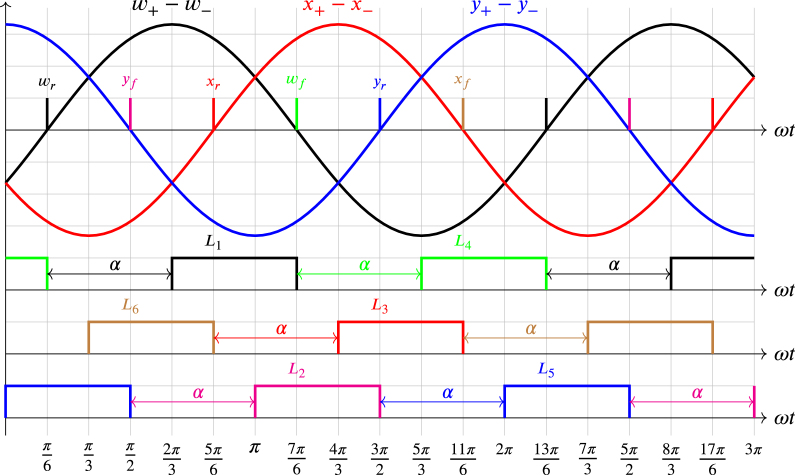
ZCDLD circuit waveforms.

The $Z_{w}$ block processes the difference of the sinusoidal signals entering terminals $w_{+}$ and $w_{-}$ to originate pulses $w_{r}$ and $w_{f}$, each time the difference between $w_{+}$ and $w_{-}$ crosses zero on the rising and falling, respectively. The $Z_{x}$ block processes the difference of the sinusoidal signals entering terminals $x_{+}$ and $x_{-}$ to produce pulses $x_{r}$ and $x_{f}$, whenever the difference between $x_{+}$ and $x_{-}$ crosses zero on the rising and falling, respectively. The $Z_{y}$ block processes the difference of the sinusoidal signals entering terminals $y_{+}$ and $y_{-}$ to generate pulses $y_{r}$ and $y_{f}$, every time the difference between $y_{+}$ and $y_{-}$ crosses zero on the rising and falling, respectively. In these signals, the subscript r refers to rising, and the subscript f refers to falling. The six impulses $w_{r}$, $w_{f}$, $x_{r}$, $x_{f}$, $y_{r}$ and $y_{f}$ enter the ports $A_{2}$, $G_{12}$, $A_{4}$, $G_{13}$, $A_{6}$ and $G_{14}$ to synchronize the signals $E_{1A},\ldots ,E_{6A}$ that enter the LED driver (LD) block, the six impulses also enter the OR block. The output s enters the port $G_{16}$ to start the analog-digital conversion of the voltage generated by the potentiometer $P_{\alpha }$, which enters through the port $A_{0}$. The potentiometer allows the variation of the delay angle α that changes between 0 and π when the variable terminal of the potentiometer changes between 0 V and $V_{cc}$, respectively. The outputs of the LD block enter the $L_{1},\ldots ,L_{6}$ terminals of the optocouplers of the three HBSGD circuits. Therefore, the LAUNCHXL-F28069M development board governs the turning on and off of the current sources in all three HBSGD circuits. It should be noted that each $L_{1},\ldots ,L_{6}$ is synchronized by its respective impulse produced by the zero crossing of the respective processed signal as is displayed in Fig. 5.

From the above, taking j=1,…,6, each $L_{j}$, and $E_{jA}$ with its synchronizing pulse and type of zero crossing of the processed signal are presented in Table 1. The symbol ↑ denotes rising zero crossing, whereas the symbol ↓ represents falling zero crossing, on the other hand, the sinusoidal entering the terminals $w_{+}$, $x_{+}$ and $y_{+}$ is a phase voltage. The sinusoidal entering the terminals $w_{-}$, $x_{-}$ and $y_{-}$, can be a phase or neutral voltage, depending on the configuration of the three HBSGD circuits.

**Table 1:** Relationship between the $E_{jA}$, $L_{j}$, and the characteristics of the processed signals.

Processed	Zero	Generated	$E_{jA}$	$L_{j}$
signal	crossing	impulse		
$w_{+}-w_{-}$	↑	$w_{r}$	$E_{1A}$	$L_{1}$
$w_{+}-w_{-}$	↓	$w_{f}$	$E_{4A}$	$L_{4}$
$x_{+}-x_{-}$	↑	$x_{r}$	$E_{3A}$	$L_{3}$
$x_{+}-x_{-}$	↓	$x_{f}$	$E_{6A}$	$L_{6}$
$y_{+}-y_{-}$	↑	$y_{r}$	$E_{5A}$	$L_{5}$
$y_{+}-y_{-}$	↓	$y_{f}$	$E_{2A}$	$L_{2}$

### 2.4 ZCDLD circuit design

**Fig. 6:**
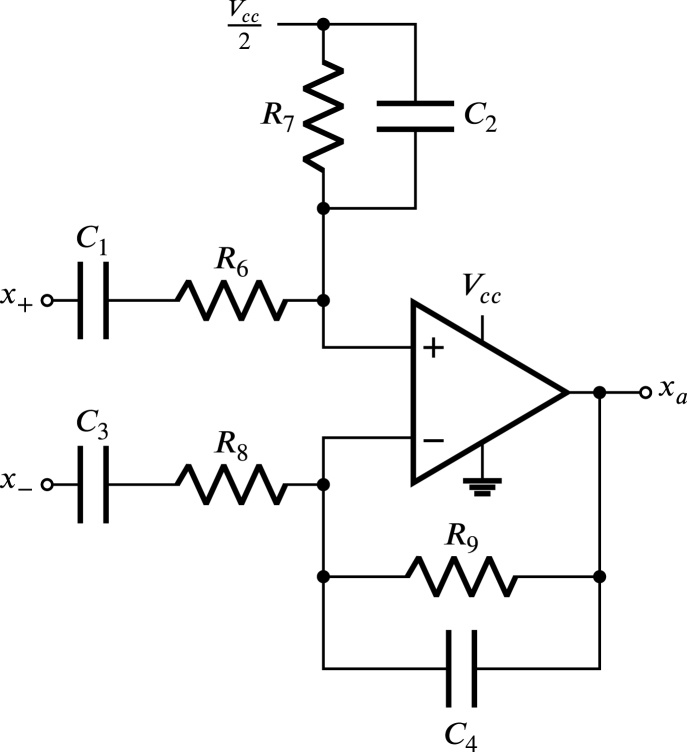
Attenuator scheme for $Z_{x}$.

Each block $Z_{w}$, $Z_{x}$ and $Z_{y}$ is composed of an attenuator, a sine-to-square signal converter and a pulse generator. In Fig. 6 the attenuator is displayed $Z_{x}$, which is an operational amplifier configured as a band-pass filter in differential mode, this filter has been selected for four main objectives: firstly, to attenuate the difference of the sinusoidal waves that enter the terminals $x_{+}$ and $x_{-}$; secondly, galvanically isolate using the dielectric barrier method to electrically separate $x_{+}$ and $x_{-}$ from ZCDLD cirquit, eliminating any physical connection between them and thus guaranteeing the electrical safety and noise reduction of the ZCDLD circuit; thirdly, reject high frequencies coming from $x_{+}$ and $x_{-}$, for the purpose of preventing malfunction or damage to the ZCDLD circuit; finally, the use of the dielectric barrier method is a more economical and efficient alternative to the use of transformers to achieve galvanic isolation. For a band-pass filter in differential mode, it is established that the resistances $R_{8}$ and $R_{6}$ are similar, just like $R_{9}$ and $R_{7}$, also the capacities $C_{3}$ and $C_{1}$ are identical, as well as $C_{4}$ and $C_{2}$.

In Fig. 8 the input signals ($x_{+}-x_{-}$) can be observed, and also the attenuator output signal ($x_{a}$), which are sinusoidal signals with a period T. Since the operational amplifier works with a single power supply the signal $x_{a}$ is displaced by a direct component, then, $x_{a}$ is given by:

(20) $$x_{a}={\overset{ˆ}{x}}_{a}+\frac{V_{cc}}{2}\;,$$

where, ${\overset{ˆ}{x}}_{a}$ corresponds to:

(21) $${\overset{ˆ}{x}}_{a}=\frac{R_{7}}{R_{6}}\frac{1}{\frac{s}{\omega_{c2}}+1}\frac{\frac{s}{\omega_{c1}}}{\frac{s}{\omega_{c1}}+1}\left(x_{+}-x_{-}\right)\;,$$

in this equation $\omega_{c1}$ and $\omega_{c2}$ are the cut-off frequencies of the high-pass and low-pass transfer functions, respectively. These cut-off frequencies determine the bandwidth of the resulting band-pass filter and are given by:

(22) $$\omega_{c1}=\frac{1}{R_{6}C_{1}}$$

(23) $$\omega_{c2}=\frac{1}{R_{7}C_{2}}\;,$$

aiming to ensuring that the frequency $\omega_{g}$ of the difference between the sinusoidal signals that enter the terminals $x_{+}$ and $x_{-}$ is within the passband of the attenuator, it is necessary to meet the conditions:

(24) $$\omega_{g}\gg \omega_{c1}\;\text{and}\;\omega_{g}\ll \omega_{c2}\;,$$

therefore, (21) can be approximated as:

(25) $${\overset{ˆ}{x}}_{a}=\frac{R_{7}}{R_{6}}\left(x_{+}-x_{-}\right)\;,$$

considering that, the maximum voltage of the difference $X_{L}$ of sinusoidal signals that enter the terminals $x_{+}$ and $x_{-}$ is equal to 300 V, the maximum voltage allowed $X_{a}$ of the signal ${\overset{ˆ}{x}}_{a}$ is equal to 1.1 V, and assuming $R_{7}$ of 1 kΩ, it can be determined $R_{6}$ from Eq. (25),

(26) $$R_{6}=\frac{X_{L}}{X_{a}}R_{7}=\text{272.73}\;\text{kΩ}\;,$$

with the objective of meeting the condition established in (24), the values of $\omega_{c1}$ and $\omega_{c2}$ are chosen equal to 3.6 rad and 10 000 rad, respectively, therefore, solving $C_{1}$ and $C_{2}$ from (22), (23) is obtained:

(27) $$C_{1}=\frac{1}{\omega_{c1}R_{6}}=\text{1}\;\text{µF}\;,$$

(28) $$C_{2}=\frac{1}{\omega_{c2}R_{7}}=\text{0.1}\;\text{µF}\;,$$

Since the configuration of the operational amplifier in the attenuator is a band-pass filter in differential mode, it is taken $R_{7}=\text{1}\;\text{kΩ}$, $R_{8}=\text{272.73}\;\text{kΩ}$, $C_{3}=\text{1}\;\text{µF}$ and $C_{4}=\text{0.1}\;\text{µF}$. The operational amplifier employed is the TLV2374 as it provides the ability to take the output from rail to rail and has a bandwidth of 3 MHz; in addition, it can operate with low supply voltage.

**Fig. 7:**
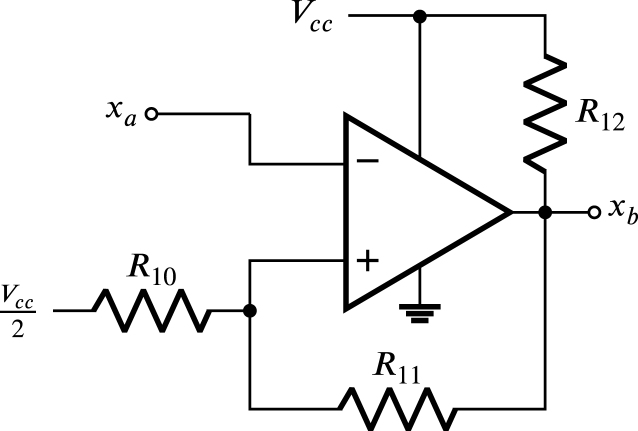
Sine-square converter circuit $Z_{x}$.

The signal $x_{a}$ is entered into the sine-square converter circuit $Z_{x}$, which is shown in Fig. 7. This converter is a comparator configured as a Schmitt trigger chosen for two main purposes: firstly to turn $x_{a}$ into a square signal, and secondly to avoid multiple transitions at the converter’s output ($x_{b}$) due to oscillations present in $x_{a}$ near the point $\frac{V_{cc}}{2}$. These oscillations are generated simultaneously by the fluctuations that appear in the difference of the sinusoidal waves that enter the terminals $x_{+}$ and $x_{-}$ near to the zero crossing point.

**Fig. 8:**
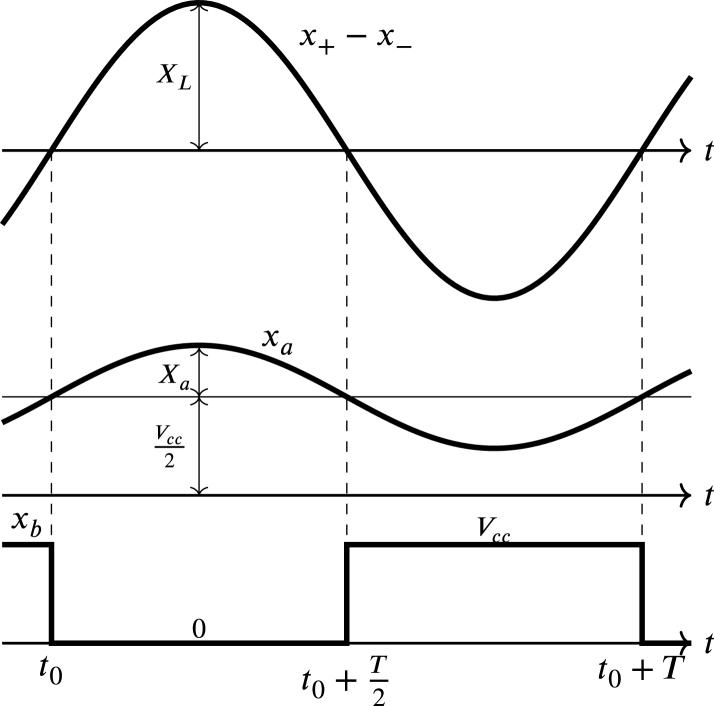
Waveforms of $x_{+}-x_{-}$, $x_{a}$ and $x_{b}$.

In this way $x_{b}$ is a square sign that varies between 0 and $V_{cc}$, as displayed in Fig. 8. The transitions in $x_{b}$ occur at low voltage threshold $V_{L}$ and high voltage $V_{H}$ given by:

(29) $$V_{L}=\frac{V_{cc}}{2}\frac{R_{11}}{R_{11}+R_{10}}\;,$$

(30) $$V_{H}=\frac{V_{cc}\left[R_{10}+\frac{1}{2}\left(R_{11}+R_{12}\right)\right]}{R_{10}+R_{11}+R_{12}}\;,$$

the transition of $V_{cc}$ to 0 in $x_{b}$ occurs when $x_{a}$ changes from its minimum value to its maximum value and reaches the value of $V_{H}$; likewise, the transition form 0 to $V_{cc}$ in $x_{b}$ occurs when $x_{a}$ changes from its maximum value to its minimum value and reaches the value of $V_{L}$. Meanwhile, the hysteresis H and the midpoint M of the hysteresis are given by:

(31) $$H=V_{H}-V_{L}$$

(32) $$M=\frac{V_{H}+V_{L}}{2}\;$$

solving $V_{L}$ and $V_{H}$ from Eqs. (31), and (32) is obtained:

(33) $$V_{L}=M-\frac{H}{2}$$

(34) $$V_{H}=M+\frac{H}{2}$$

Considering that fluctuations occur in the difference of the sinusoidal waves that enter the terminals $x_{+}$ and $x_{-}$near the zero crossing point, with maximum amplitude of approximately 2% of the maximum value of the line voltage, it is selected H equal to 40 mV. Likewise, assuming M with the value $\frac{V_{cc}}{2}$ and substituting in Eqs. (33), and (34) the values of $V_{L}$ and $V_{H}$ are equal to 1.63 V and 1.67 V respectively. Since these values are very close to $\frac{V_{cc}}{2}$, it can be stated that the down and up transitions in signal $x_{b}$ shown in Fig. 8 occur approximately in $t_{0}$ and $t_{0}+\frac{T}{2}$ respectively. Finally, choosing $R_{10}$ equal to 1.8 kΩ and solving (29), (30), the values of $R_{11}$ and $R_{12}$ are equal to 150 kΩ and 1.5 kΩ. The comparator integrated circuit used for the sine to square signal converter is the LM339, which is low cost, offers the ability to operate with low supply voltage, and is easy to purchase in electronic component stores and distributors.

**Fig. 9:**
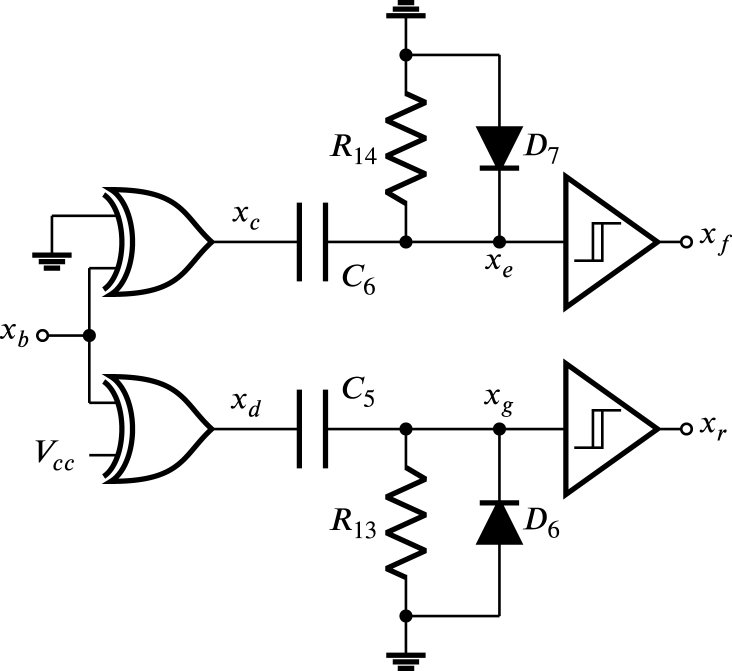
Impulse generator of $Z_{x}$.

In Fig. 9 the impulse generator $Z_{x}$ is shown composed of two XOR gates, two high-pass networks with diode and two buffer circuits with Schmitt trigger, where the signal $x_{b}$ is the input. The upper XOR gate acts as a buffer, while the lower XOR gate acts as a negator, so, $x_{c}$ and $x_{d}$ are square signals that enter their corresponding high-pass network, originating the impulses $x_{e}$ and $x_{g}$ respectively. The diodes $D_{6}$ and $D_{7}$ of the RC networks prevent negative excursions in these impulses. The impulses $x_{e}$ and $x_{g}$ that are not square are processed by their corresponding buffer with a Schmitt trigger to transform them into square impulses $x_{f}$ and $x_{r}$ respectively. The two high-pass networks with diode are similar, for this reason, the values of their resistors and capacitors are identical as well as the commercial references of their diodes; on the other hand, each buffer is implemented using two inverters in cascade with Schmitt trigger.

**Fig. 10:**
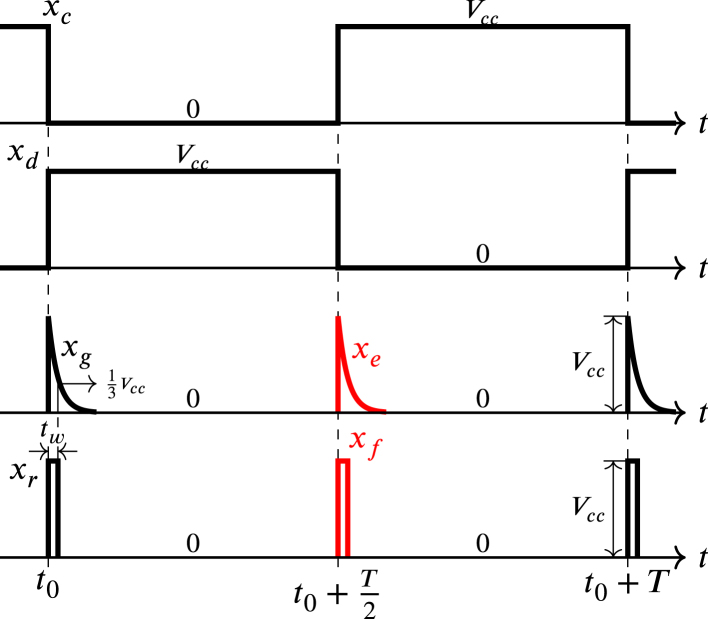
Waveforms of $x_{c}$, $x_{d}$, $x_{g}$, $x_{e}$, $x_{r}$, and $x_{f}$.

Regarding Fig. 8, Fig. 10, during the positive half-cycle of the sinusoidal $x_{+}-x_{-}$, the signal $x_{c}$ is held at 0, while the signal $x_{d}$ is maintained at $V_{cc}$. Conversely, during the negative half-cycle of the sinusoidal $x_{+}-x_{-}$, the opposite occurs, where the signal $x_{c}$ is set to $V_{cc}$, while the signal $x_{d}$ is set to 0. The $x_{e}$ and $x_{g}$ impulses are generated when a transition from 0 to $V_{cc}$ occurs in $x_{c}$ and $x_{d}$, respectively. These impulses are given by:

(35) $$x_{f}=\left\{\begin{array}{cc} 0 & t_{0}\leq t<t_{0}+\frac{T}{2}\\ V_{cc}e^{-\frac{t-t_{0}-\frac{T}{2}}{\tau }} & t_{0}+\frac{T}{2}\leq t<t_{0}+T \end{array}\right)\;,$$

(36) $$x_{g}=\left\{\begin{array}{cc} V_{cc}e^{-\frac{t-t_{0}}{\tau }} & t_{0}\leq t\leq t_{0}+\frac{T}{2}\\ 0 & t_{0}+\frac{T}{2}<t<t_{0}+T \end{array}\right)\;,$$

where the value of $V_{cc}$ is equal to 3.3 V and the time constant is given by:

(37) $$\tau =R_{13}C_{5}\;,$$

In Fig. 10, the transition from 0 to $V_{cc}$ in signal $x_{r}$ occurs when $x_{g}$ changes from 0 to $V_{cc}$ and reaches the value of $\frac{2}{3}V_{cc}$, however, since this change is overly fast, the transitions from 0 to $V_{cc}$ in both $x_{r}$ and $x_{g}$ occurs almost simultaneously at $t_{0}$; likewise, the transition from $V_{cc}$ to 0 in signal $x_{r}$ occurs in $t_{0}+t_{w}$ when $x_{g}$ decreases exponentially from $V_{cc}$ to 0 and reaches the value of $\frac{1}{3}V_{cc}$. Transitions in $x_{f}$ occur in the same way in relation to the variations in signal $x_{e}$.

According to datasheet for the LAUNCHXL-F28069M development board, the cycle time (SYSCLKOUT) is 11.11 ns, for the operating frequency of 90 MHz. As mentioned previously, the signals $E_{1A}-E_{6A}$ are synchronized using impulses $w_{r}$, $w_{f}$, $x_{r}$, $x_{f}$, $y_{r}$, and $y_{f}$. According to the datasheet, the minimum required width of these pulses is twice SYSCLKOUT, therefore, the pulse width time $t_{w}$ must be much larger than the minimum pulse width, namely:

(38) $$t_{w}\gg \text{22.22}\;\text{ns}$$

then, selecting $t_{w}$ equal to 110 µs that meets the condition (38). Furthermore, according to Fig. 10 when evaluating Eq. (36) in $t_{0}+t_{w}$ is obtained:

(39) $$x_{g}\left(t_{0}+t_{w}\right)=V_{cc}e^{-\frac{t_{w}}{\tau }}=\frac{1}{3}V_{cc}\;,$$

solving τ from Eq. (39), is obtained:

(40) $$\tau =\text{0.91}\;t_{w}=R_{13}C_{5}\;,$$

choosing $C_{5}$ equal to 10 nF, and calculating $R_{13}$ from Eq. (40) is found that:

(41) $$R_{13}=\text{0.91}\;\frac{t_{w}}{C_{5}}=\text{10}\;\text{kΩ}\;,$$

since both high-pass networks are identical, then, $C_{6}=\text{10}\;\text{nF}$ and $R_{14}=\text{10}\;\text{kΩ}$. Meanwhile the XOR gate corresponds to the integrated circuit 74HC86. The inverter circuit to implement the buffer with Schmitt trigger is the CD40106, and the diode 1N4148 is selected for $D_{6}$, and $D_{7}$. These components are low cost and easy to purchase at electronic component stores and distributors.

The attenuator, the sine-to-square signal converter, and pulse generator within blocks $Z_{w}$ and $Z_{y}$ operate in the same way as in block $Z_{x}$ (above explained), thus generating impulses $w_{r}$, $w_{f}$, $y_{r}$, and $y_{f}$.

**Fig. 11:**
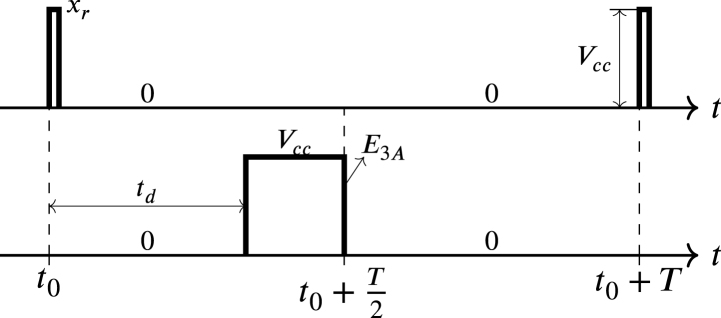
Waveforms of $x_{r}$ and $E_{3A}$.

According to Table 1, each signal $L_{1},\ldots ,L_{6}$ is synchronized with its respective impulse; in the case of the $E_{3A}$ is the impulse $x_{r}$. Regarding Fig. 11, at $t_{0}$ a transition from 0 to $V_{cc}$ occurs in $x_{r}$ initiating the delay time ($t_{d}$). During $t_{d}$, $E_{3A}$ remains at 0. At the end of $t_{d}$, $E_{3A}$ transitions from 0 to $V_{cc}$, then holds at $V_{cc}$ until $t_{0}+\frac{T}{2}$. Exactly in $t_{0}+\frac{T}{2}$ signal $E_{3A}$ transitions from $V_{cc}$ to 0 remaining in 0 until $t_{0}+T$, when another transition from 0 to $V_{cc}$ occurs in signal $x_{r}$, in this way $t_{d}$ starts again.

The delay angle α is related to the delay time $t_{d}$ by the form:

(42) $$\alpha =\frac{2\pi \;t_{d}}{T}\;,$$

where T is the period of the sinusoidal signal $x_{+}-x_{-}$. On the other hand, the minimum and maximum values of $t_{d}$ are equal to 0 and $\frac{T}{2}$ respectively; therefore, the angular interval of α is defined by:

(43) 0≤α≤π,

The signals $E_{1A}$, $E_{2A}$, $E_{4A}$, $E_{5A}$, and $E_{6A}$ work in a similar way as described above and their waveforms have amplitude and pulse width similar to the waveform of $E_{3A}$ in Fig. 11; however, they are displaced due to the respective impulses that synchronize them, as indicated in Table 1.

**Fig. 12:**
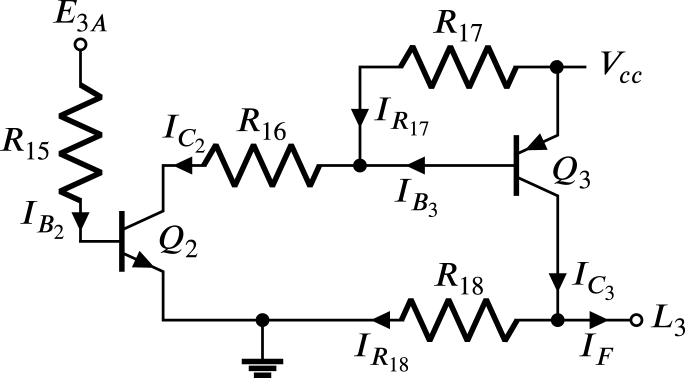
Circuit to boost $E_{3A}$ current.

The maximum current of the six $E_{1A},\ldots ,E_{6A}$ in source mode is 4 mA. However, in Section 2.2, it is established that the $I_{F}$ in the HBSGD circuit is 6.8 mA. For this reason, the LD block is required, which consists of six circuits, each responsible for boosting the current of the respective signals $E_{1A},\ldots ,E_{6A}$. Fig. 12 displays the circuit that reinforces the current of $E_{3A}$. The bipolar transistor $Q_{2}$ corresponds to 2N2222, and the bipolar transistor $Q_{3}$ is the 2N2907. These transistors are low cost and easy to purchase in electronic component stores and distributors. Since $Q_{1}$ and $Q_{2}$ are complementary bipolar transistors, their minimum forward current gains $h_{FE_{2}}$, and $h_{FE_{3}}$ are identical and equal to 40. To simplify the analysis, the saturation collector-to-emitter voltages on both transistors approach zero.

On the other hand, $R_{18}$ ensures a voltage of 0 V at terminal $L_{3}$ when $Q_{3}$ is in the cut-off region, while $R_{17}$ maintains an emitter-to-base voltage in $Q_{3}$ below the threshold to ensure it remains in the cut-off region when $Q_{2}$ is also in cut-off region. $R_{18}$ is selected as 1 kΩ; hence, the current flowing through $R_{18}$ is given by:

(44) $$I_{R_{18}}=\frac{V_{cc}}{R_{18}}=\text{3.3}\;\text{mA}\;,$$

in this way, the collector current $Q_{3}$ corresponds to:

(45) $$I_{C_{3}}=I_{F}+I_{R_{18}}=\text{10.1}\;\text{mA}\;,$$

to guarantee reduced voltages collector-to-emitter in $Q_{2}$, and emitter-to-collector in $Q_{3}$ when these transistors are in the saturation region, it is required to meet the following conditions:

(46) $$\beta_{f_{2}}<h_{FE_{2}}\;,$$

(47) $$\beta_{f_{3}}<h_{FE_{3}}\;,$$

where $\beta_{f_{2}}$ and $\beta_{f_{3}}$ are the minimum direct current forced gains of $Q_{2}$ and $Q_{3}$ respectively. Assuming a $\beta_{f_{3}}$ equal to 25 that meets condition (47), the base current of $Q_{3}$ is given by:

(48) $$I_{B_{3}}=\frac{I_{C_{3}}}{\beta_{f_{3}}}=\text{404}\;\text{µA}\;,$$

choosing $R_{17}$ to be 4.7 kΩ and assuming a $V_{EB_{3}}$ of 0.7 V, and regarding Fig. 12, the collector current in $Q_{2}$ is given by:

(49) $$I_{C_{2}}=\frac{V_{EB_{3}}}{R_{17}}+I_{B_{3}}=\text{553}\;\text{µA}\;,$$

in this way the resistance $R_{16}$ is given by:

(50) $$R_{16}=\frac{V_{cc}-V_{EB_{3}}}{I_{C_{2}}}=\text{4.7}\;\text{kΩ}\;,$$

In order to maintain the base-to-emitter voltage of $Q_{2}$ below the threshold (to ensure that $Q_{2}$ is in the cut-off region), it is necessary that $R_{15}$ is not overly high; in this way, taking a $\beta_{f_{2}}$ equal to 2.13 the condition (46) is met producing a base current in $Q_{2}$ defined by:

(51) $$I_{B_{2}}=\frac{I_{C_{2}}}{\beta_{f_{2}}}=\text{259.6}\;\text{µA}\;,$$

assuming a $V_{BE_{2}}$ equal to 0.7 V, the resistance $R_{15}$ is given by:

(52) $$R_{15}=\frac{V_{cc}-V_{BE_{2}}}{I_{B_{2}}}=\text{10}\;\text{kΩ}$$

the resistances $R_{6}$ to $R_{18}$ are chosen to dissipate a maximum power of 0.25 W, the capacitors $C_{1}$ to $C_{6}$ can be ceramic or polyester, the semiconductor devices $D_{6}$, $D_{7}$, $Q_{2}$, and $Q_{3}$ operate with voltages and currents that are within the ranges specified by their corresponding datasheets.

## 3 Design files summary

The Gerber files corresponding to the PCBs, the projects in Altium, and the schematic diagrams of the HBSGD and ZCDLD circuits are available to implement the experimental SCRs module. Additionally, the source codes related to the LAUNCHXL-F28069M development board are provided.

**Table:** 

Design filename	File type	Open source license	Location of the file
SCRs_experimental_module	.C	CC-BY-NC 4.0	https://doi.org/10.17632/vfsj3zhsgf.2
microprocessor_initialization	.C	CC-BY-NC 4.0	https://doi.org/10.17632/vfsj3zhsgf.2
ZCDLDschematic	.PDF	CC-BY-NC 4.0	https://doi.org/10.17632/vfsj3zhsgf.2
HBSGDschematic	.PDF	CC-BY-NC 4.0	https://doi.org/10.17632/vfsj3zhsgf.2
ZCDLDgerbers	.ZIP	CC-BY-NC 4.0	https://doi.org/10.17632/vfsj3zhsgf.2
HBSGDgerbers	.ZIP	CC-BY-NC 4.0	https://doi.org/10.17632/vfsj3zhsgf.2
ZCDLDproject	.ZIP	CC-BY-NC 4.0	https://doi.org/10.17632/vfsj3zhsgf.2
HBSGDproject	.ZIP	CC-BY-NC 4.0	https://doi.org/10.17632/vfsj3zhsgf.2
ZCDLDcomponentlist	.XLSX	CC-BY-NC 4.0	https://doi.org/10.17632/vfsj3zhsgf.2
HBSGDcomponentlist	.XLSX	CC-BY-NC 4.0	https://doi.org/10.17632/vfsj3zhsgf.2
ANOTHERcomponentlist	.XLSX	CC-BY-NC 4.0	https://doi.org/10.17632/vfsj3zhsgf.2

The description of the above mentioned files is as follows:

• **SCRs_experimental_module**: Main code that runs on the development board microprocessor LAUNCHXL-F28069M.
• **microprocessor_initialization**: Code to set clocks PWMs, ADC, and comparators, as well as the microprocessor’s interrupt vector.
• **ZCDLDschematic**: Electrical circuit diagram ZCDLD implemented in the PCB.
• **HBSGDschematic**: Electrical circuit diagram HBSGD implemented in the PCB.
• **ZCDLDgerbers**: ZIP archive with the PCB Gerber files for circuit ZCDLD.
• **HBSGDgerbers**: ZIP archive with the PCB Gerber files for circuit HBSGD.
• **ZCDLDproject**: ZIP archive with the PCB project files in Altium for circuit ZCDLD.
• **HBSGDproject**: ZIP archive with the PCB project files in Altium for circuit HBSGD.
• **ZCDLDcomponentlist**: List of electronic circuit components ZCDLD.
• **HBSGDcomponentlist**: List of electronic circuit components HBSGD.
• **ANOTHERcomponentlist**: List of other components external to the circuits ZCDLD, and HBSGD.

## 4 Bill of materials summary

The list of components is separated into three parts, namely, components for the HBSGD circuit, components for the ZCDLD circuit, and other additional components. Complete lists of materials are available in the repository [12].

## 5 Build instructions

This section describes the build instructions, in the first part general guidelines are addressed; then the interconnection of the HBSGD and ZCDLD circuits and finally the implementation of the PCBs circuits.

### 5.1 General guidelines

Printed Circuit Boards (PCBs) can be manufactured by a company specialized in prototyping and assembly, by sending the Gerber files available in the repository [12]. The assembly of the components on the PCBs can be done manually with care, using standard assembly techniques and appropriate tools, such as a soldering iron with a temperature regulator, high-quality solder and a PCB holder.

The interconnection between the three HBSGD circuits, the ZCDLD circuit, the three power diodes, the seven 5 V power supplies, as well as other components of the module are described in Section 5.2.

It is important to prevent high current leads from the HBSGDs from crossing over the ZCDLD circuit as this may cause malfunctions due to Electromagnetic Interference (EMI). Since the potentiometer is located outside the ZCDLD circuit, the line carrying the voltage to $A_{0}$ to vary α may be susceptible to EMI, which could cause malfunctions in the ZCDLD. Therefore, the potentiometer leads must be shielded to minimize this EMI, and they must be kept away from the HBSGDs and their lead (connection) wires.

### 5.2 Interconnection of the HBSGD and ZCDLD circuits

**Fig. 13:**
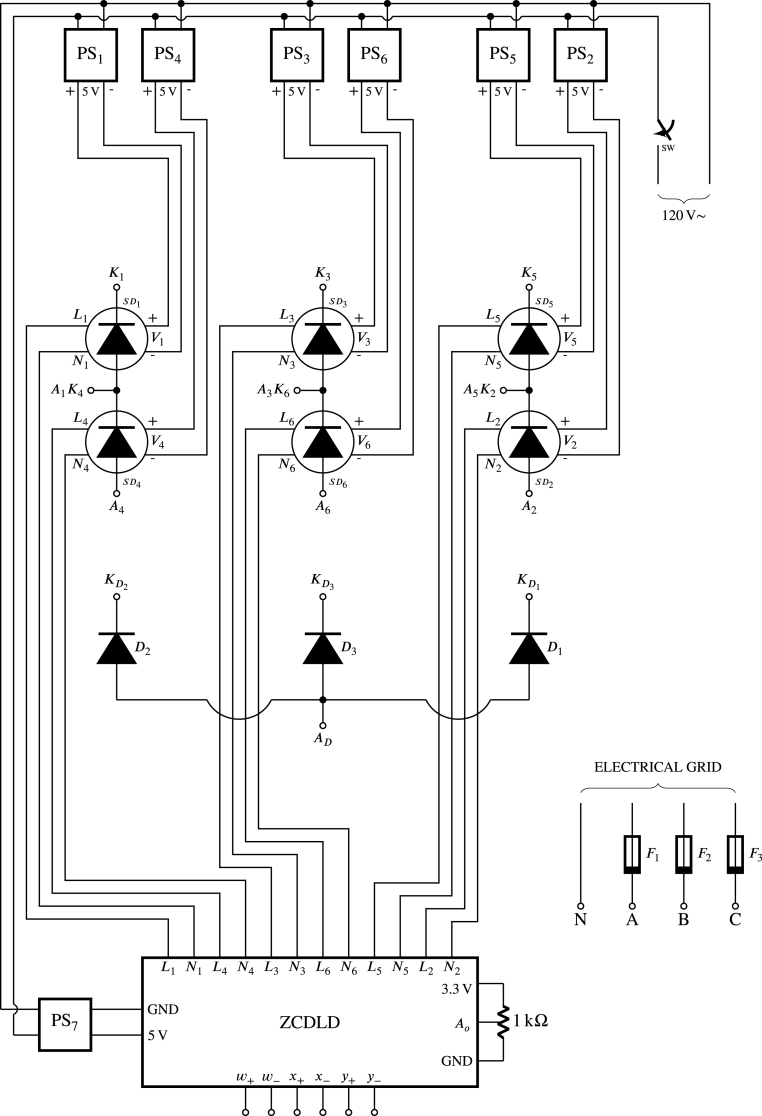
Electrical diagram of the experimental SCR module.

In Fig. 13, the electrical diagram of the proposed experimental module is shown as composed of three HBSGD, one ZCDLD, three power diodes, and seven power supplies of 5 V (this last is necessary as each gate driver needs an isolated power supply). As can be seen $SD_{1}$ and $SD_{4}$ are join together to form ${\text{HBSGD}}_{1}$, connecting the anode $SD_{1}$ with the cathode of $SD_{4}$, producing the terminal $A_{1}K_{4}$. The cathode of $SD_{1}$ is designated as terminal $K_{1}$ and the anode of $SD_{4}$ is called terminal $A_{4}$. Meanwhile $SD_{3}$ and $SD_{6}$ are join together to create ${\text{HBSGD}}_{2}$, connecting the anode of $SD_{3}$ with the cathode of $SD_{6}$, which produces the terminal $A_{3}K_{6}$. The cathode of $SD_{3}$ is designated as terminal $K_{3}$ and the anode of $SD_{6}$ is labeled as terminal $A_{6}$. In the same way $SD_{5}$ and $SD_{2}$ are employed to form ${\text{HBSGD}}_{3}$, connecting the anode of $SD_{5}$ with the cathode of $SD_{2}$, creating the terminal $A_{5}K_{2}$, the cathode of $SD_{5}$ is designated as terminal $K_{5}$ and the anode $SD_{2}$ is called terminal $A_{2}$. Consequently, the module proposed in this work allows configuring rectifiers and controllers that can be three-phase. Besides the anode connection of $D_{1}$, $D_{2}$, and $D_{3}$ produces the terminal $A_{D}$, while the cathodes of $D_{1}$, $D_{2}$, and $D_{3}$ are designated as the terminals $K_{D_{1}}$, $K_{D_{2}}$, and $K_{D_{3}}$ respectively; these three power diodes allow configuring single-phase, two-phase or three-phase half-wave phase control rectifiers. The phases of the electrical network enter the module through the fuses $F_{1}$, $F_{2}$, and $F_{3}$, which provide protection against overloads or short circuits. These three fuses have a blowing current value of 3 A. Additionally, the reference of these phases enters directly into the module, therefore, in terminals A, B, C and N there are the three-phases and the reference of the electrical network, to be connected to the circuits ${\text{HBSGD}}_{1}$, ${\text{HBSGD}}_{2}$, ${\text{HBSGD}}_{3}$, and ZCDLD. The previously mentioned terminals together with the terminals $w_{+}$, $w_{-}$, $x_{+}$, $x_{-}$, $y_{+}$, and $y_{-}$ are female banana type.

The power supplies of $SD_{1}$ to $SD_{6}$ must be galvanically isolated from each other to avoid short circuits between the terminals $K_{1}$, $K_{3}$, $K_{5}$, $A_{1}K_{4}$, $A_{3}K_{6}$, and $A_{5}K_{2}$, since these terminals correspond to the cathodes of the SCRs electrically separated (see Fig. 13) and connected to the negative pole of its corresponding power supply (see Fig. 3). Also, the ZCDLD power supply is required to be galvanically isolated from other sources to ensure electrical safety and reduce noise in ZCDLD. For these reasons, ${\text{PS}}_{1}$ to ${\text{PS}}_{7}$ are galvanically isolated sources that produce a DC voltage of 5 V from an alternating voltage of 120 V, which supply power to the circuits ${\text{HBSGD}}_{1}$, ${\text{HBSGD}}_{2}$, ${\text{HBSGD}}_{3}$ and ZCDLD; these sources are mobile phone battery chargers due to their wide use and low cost. Besides $L_{1}$-$L_{6}$ are connected through a resistor to the anodes of the light-emitting diodes of the optocouplers $SD_{1}$-$SD_{6}$ respectively (see Fig. 3); these signals have the same waveforms $E_{1A}$-$E_{6A}$, even though $L_{1}$-$L_{6}$ can handle a higher current (in source or sink mode) compared to $E_{1A}$-$E_{6A}$. In addition $N_{1}$-$N_{6}$ are connected to the cathodes of the emitting diodes of the optocouplers $SD_{1}$-$SD_{6}$ respectively, as well as the internal reference of the power supply of the ZCDLD (GND). The potentiometer is used to vary the pulse width of $E_{1A}$-$E_{6A}$, which modifies the value of $t_{d}$ that according to Eq. (42) is directly proportional to α. This potentiometer is a multiturn type, since it allows highly precise adjustments of α given by the several full turns of the shaft to go through the full resistance range, it also keeps its resistance setting stable despite changes in temperature, vibration, and other environmental factors.

**Fig. 14:**
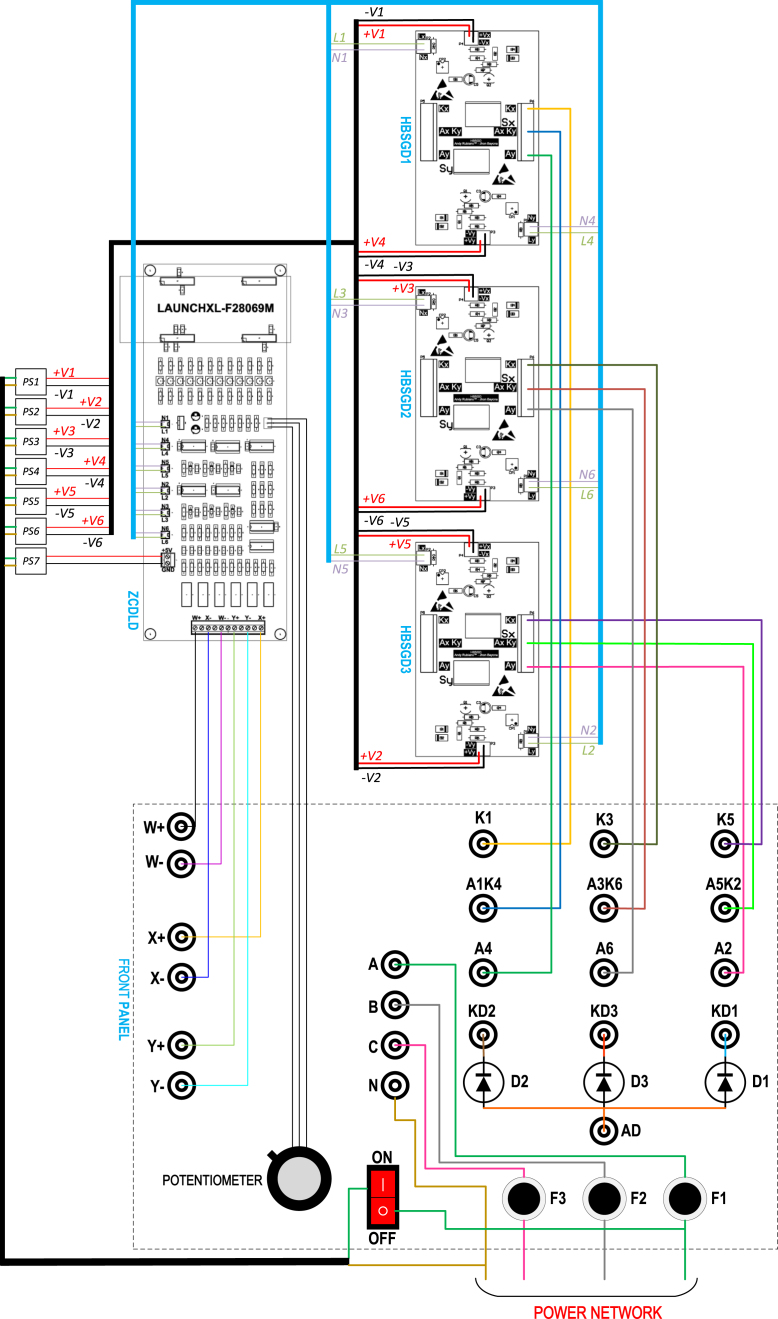
Assembly of the PCBs, power supplies, and front panel of the SCR module.

In the module, the conductors are grouped into two categories (based on the RMS current they carry) to avoid using multiple sizes according to the American Wire Gauge (AWG) standard. The high RMS current conductors are AWG18 and connect to the terminals $K_{1}$, $K_{3}$, $K_{5}$, $A_{1}K_{4}$, $A_{3}K_{6}$, $A_{5}K_{2}$, $A_{2}$, $A_{4}$, $A_{6}$, $K_{D_{1}}$, $K_{D_{2}}$, $K_{D_{3}}$, $A_{D}$, A, B, C, and N; likewise, they (current conductors) carry the three-phase electrical network to the module. On the other hand, the low RMS current conductors are AWG24 and attached to the terminals $w_{+}$, $w_{-}$, $x_{+}$, $x_{-}$, $y_{+}$, $y_{-}$, $L_{1}$-$L_{6}$, and $N_{1}$-$N_{6}$, also, connect the potentiometer with ZCDLD and the direct current lines of the sources ${\text{PS}}_{1}$-${\text{PS}}_{7}$ with ${\text{HBSGD}}_{1}$, ${\text{HBSGD}}_{2}$, ${\text{HBSGD}}_{3}$, and ZCDLD. In addition, the AWG18 conductors are employed to configure the module as a phase control rectifier or AC–AC voltage controller (whether three-phase, two-phase or single-phase). Both ends of these conductors have male banana terminals. Additionally, Fig. 14 displays the connections in detail to facilitate the successful module assembly. It shows the connectors with their respective labels on the PCBs of ${\text{HBSGD}}_{1}$, ${\text{HBSGD}}_{2}$, ${\text{HBSGD}}_{3}$, and the ZCDLD, as well as a suggested layout of the PCBs, power supplies, female banana plugs, power diodes, potentiometer, switch, and the three protection fuses.

**Fig. 15:**
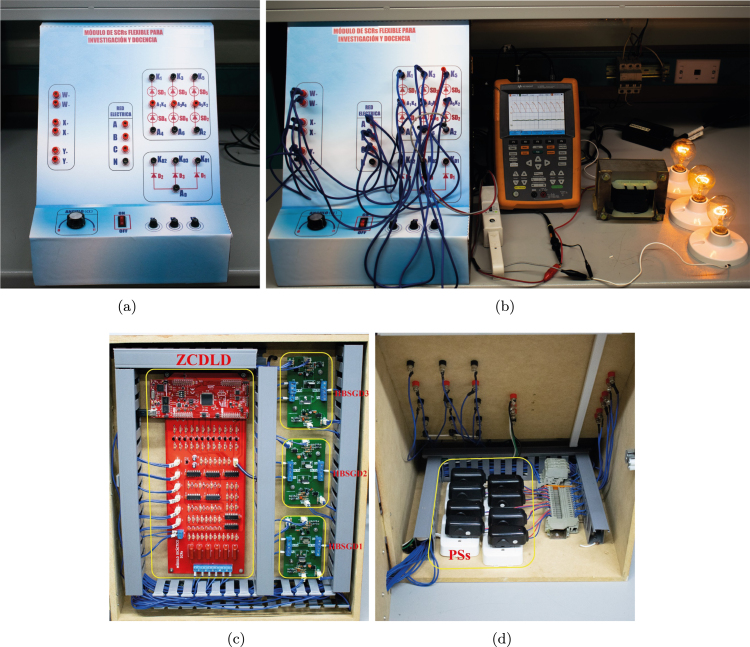
(a) Module configurated as three-phase rectifier. (b) Front panel. (c) Circuits ${\text{HBSGD}}_{\text{s}}$, and ZCDLD. (d) Module power supplies.

Finally, in Fig. 15(a), the female banana connectors on the front panel of the module can be observed. Fig. 15(b) displays the module connected as a three-phase full wave phase control rectifier using the conductors with male banana type terminals; likewise, Fig. 15, Fig. 15 shows the location of the circuits ${\text{HBSGD}}_{1}$, ${\text{HBSGD}}_{2}$, ${\text{HBSGD}}_{3}$, and ZCDLD, as well as the power supplies inside the module. If a cabinet is not available, the module components can be distributed on an acrylic or wooden base.

## 6 Operation instructions

This section provides the procedure for setting and operating the module displaying the general way to carry out a practice (experiment). In the validation and characterization section, the development of different experiments that can be carried out with the module is described. It should be noted that this module was designed to be easy for students and researchers. The following are the steps to operate the module:

1. Place the teaching module in a suitable space (workbench) where power is available for operation.
2. Prepare the connectors, measurement devices, and the respective loads to be used.
3. Set the experiment by selecting and performing the type of connection for the SCRs on the front panel.
4. Power up the device and verify the correct operation of the experiment performed.
5. Verify the correct operation of the experiment and carry out the respective measurements and data acquisition.
6. To finish the experiment, the module must be turned off, the connectors, measuring elements, and loads removed and placed in their respective storage space.

Finally, it is to point out that the module’s front panel is properly labeled, which facilitates the identification of the connections to be made for both students and researchers.

## 7 Validation and characterization

The SCR module proposed in this document eliminates the need for trainees (students) to build circuits such as phase control rectifiers and AC–AC voltage controllers typically included in power electronics lectures (subjects) in electrical engineering and electronics. This reduces the time and costs associated with component selection and design of Printed Circuit Boards (PCBs), making it easier for learners to study and understand these circuits. In this work, five laboratory experiments were carried out to demonstrate the usefulness and versatility of the SCR module in the implementation of phase control rectifiers, and AC–AC voltage controllers, under various load conditions. In addition, these five experiments were implemented in simulation software to validate the results achieved with the module. The simulation software employed was MATLAB 2018b using “*simulink*” and the toolbox of “*simscape*” and “*simscape electrical*”.

In the first experiment, it is implemented a Three-Phase Full Wave Rectifier (TPFWR), where the load consists of a resistance and inductance in series. The second proposed experiment is essential to understand the functioning of the Direct Current Transmission System (DCTS). The third experiment corresponds to a Three-Phase Half Wave Rectifier (TPHWR) with resistive load. The fourth experiment consists of a Three-Phase Voltage Controller (TPVC) AC–AC with a resistive load arranged in a star configuration. The fifth experiment is relevant to understand the operation of a Reactive Power Static Compensator (RPSCs), employed to keep the power factor close to unity. The parameter α is significant in all five experiments; however, direct measurement of α with an oscilloscope is not possible. To overcome this drawback, α is determined using $t_{d}$ in Eq. (42). Parameter $t_{d}$ is the time interval between the transition from 0 to $V_{cc}$ of the generated impulse and the transition from 0 to $V_{cc}$ in the respective terminal $L_{j}$ (see Table 1).

### 7.1 First experiment: TPFWR with R-L load

**Fig. 16:**
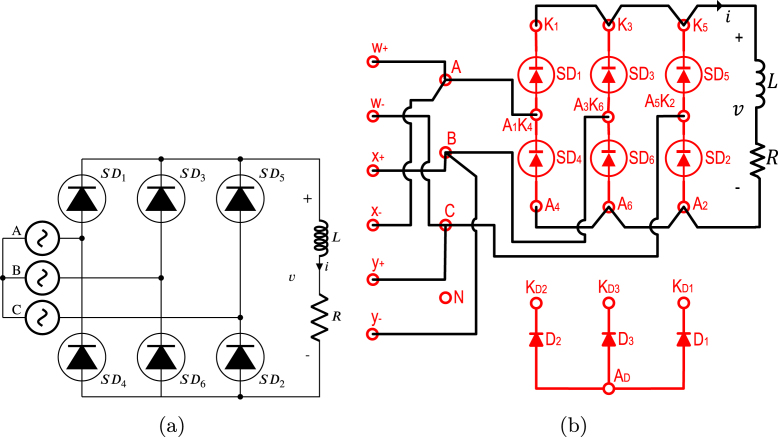
TPFWR with load R-L. (a) Simplified schematic diagram. (b) Connections on the front panel of the module.

**Fig. 17:**
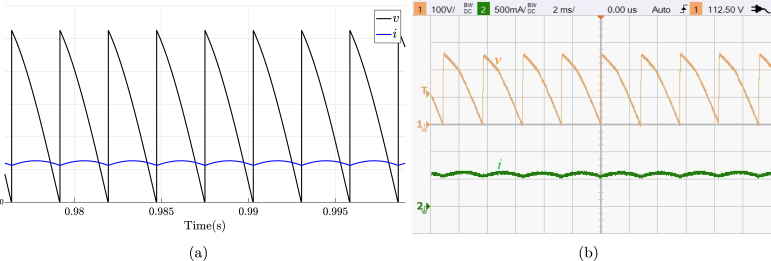
Waveforms for v, and i for TPFWR with R-L load. (a) Simulation; v: 50 V/div, i: 0.5 A/div. (b) Experimental results.

TPFWR is an AC–DC converter circuit employed in various implementations ranging from small rectifiers to large high voltage direct current transmissions. The circuit is also called a six-pulse phase-controlled rectifier since the output voltage waveform shows six pulses per line period. The TPFWR is used for motor drives, electrochemical processes, controlled power supplies, traction equipment, among others. This subsection shows the use of the proposed SCR module in an electrical or electronic engineering laboratory activity, which consists of connecting a resistance and inductance in series to the output of a TPFWR, as illustrated in Fig. 16(a). The corresponding connections required for its implementation on the SCRs module are shown in Fig. 16(b). The parameters used in this implementation are the following: RMS voltage of 125 V for the power network (A,B,C), a resistance R equal to 225 Ω, an inductance L of 1.3 H with an equivalent series resistance of 10 Ω due to losses in copper, and α equal to $\frac{\pi }{3}$. The activity begins energizing the module power supply, followed by setting the desired value for α; finally the module connects to the power network (A,B,C). Fig. 17 displays the current waveforms i and the voltage v in the load, obtained both in simulation and experimentally in the SCRs module. The functionality of the module was checked by comparing the experimental waveforms with the simulated ones. The results show a significant agreement, which allows affirming the effectiveness of the module in this experiment.

### 7.2 Second experiment: DCST implementation

**Fig. 18:**
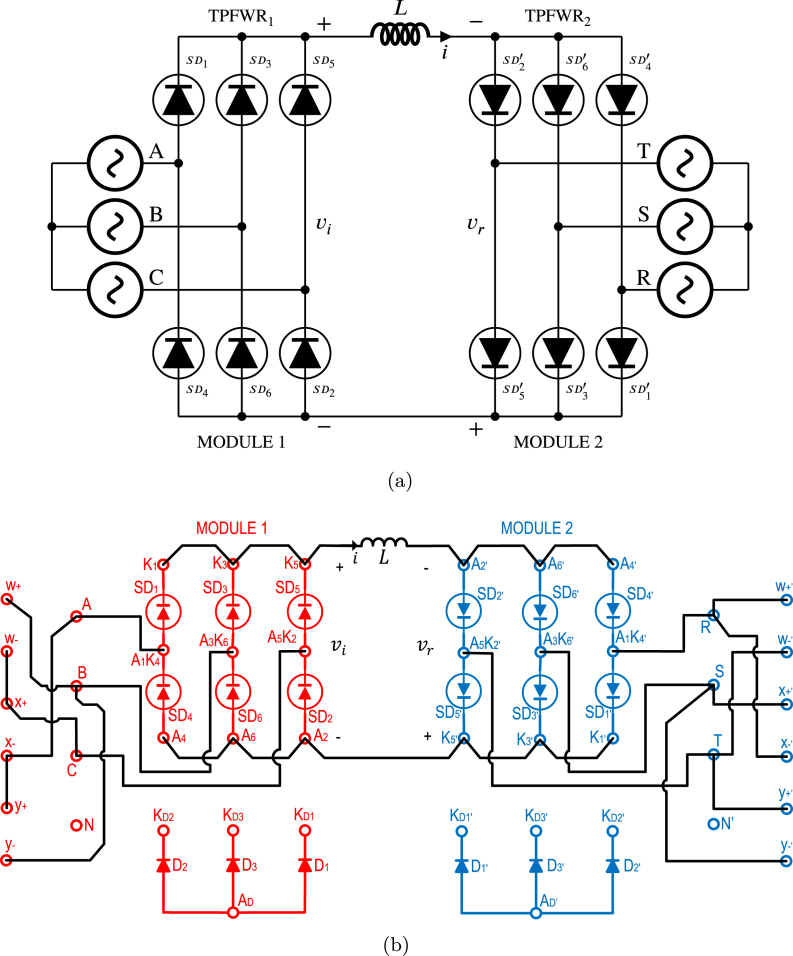
DCST circuit implementation. (a) Simplified schematic diagram. (b) Connections on the front panels of the two modules.

**Fig. 19:**
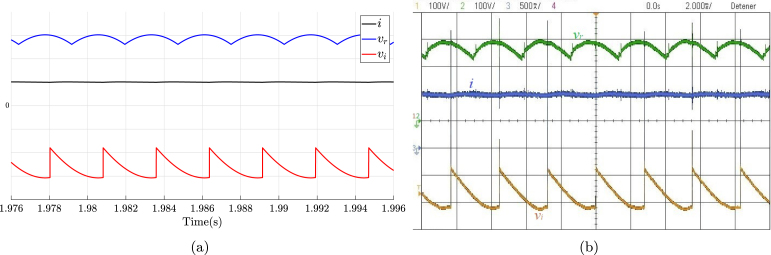
Waveforms for $v_{r}$, $v_{i}$, and i for DCST. (a) Simulation for $v_{r}$, and $v_{i}$: 100 V/div, i: 1 A/div. (b) Experimental results.

The DCST is a system used in direct current transmission lines that allows electrical power transmission over long distances. This consists of two TPFWRs interconnected back to back through an inductance as shown in Fig. 18(a). The angles α, and β corresponding to ${\text{TPFWR}}_{1}$, and ${\text{TPFWR}}_{2}$, govern the magnitude and direction of power flow in both TPFWRs. When α is located in the range of $\frac{\pi }{2}$ to π, and β from 0 to $\frac{\pi }{2}$, the ${\text{TPFWR}}_{1}$ works as an inverter while ${\text{TPFWR}}_{2}$ as a rectifier, causing a flow of power from the system (R,S,T) to the system (A,B,C). On the other hand, when α is located in the range of 0 to $\frac{\pi }{2}$, and β from $\frac{\pi }{2}$ to π, the ${\text{TPFWR}}_{1}$ operates as a rectifier while ${\text{TPFWR}}_{2}$ as an inverter, causing a power flow from the system (A,B,C) to the system (R,S,T). In conclusion, the electrical grid connected in the input of the TPFWR works as a rectifier supplies power, and the electrical grid connected in the output of the TPFWR operates as an inverter that absorbs this power. This subsection displays the application of the proposed SCR module in an electrical or electronic engineering laboratory activity. The purpose of this activity is to transfer electrical power from the system (R,S,T) to the system (A,B,C) by using the DCST, in this way, Fig. 18(b) shows the necessary connections for its implementation on the two SCR modules. In this activity, the following parameters were used: RMS voltage of 125 V for the grids (A,B,C), and (R,S,T), an inductance L equal to 1.3 H with an equivalent series resistance of 10 Ω due to losses in the copper, and an average current of 1 A in the inductance. To carry out this activity; first, the power supplies of both modules are energized; second, β is set to 0, and α to π; third, module 1 is connected to the grid (A,B,C) and module 2 to the grid (R,S,T); finally, α is reduced until the transferred power or desired average current over the inductance is reached. The operation of the SCR module was corroborated by comparing the experimental waveforms with the simulated ones. Fig. 19 shows inductance current waveforms i, and the output voltages of the TPFWRs $v_{i}$, and $v_{r}$, obtained both in simulation and experimentally in the module, in this way, there is a notable coherence between the experimental and simulated results, ratifying the validity of the module in this experiment.

### 7.3 Third experiment: TPHWR with R load

**Fig. 20:**
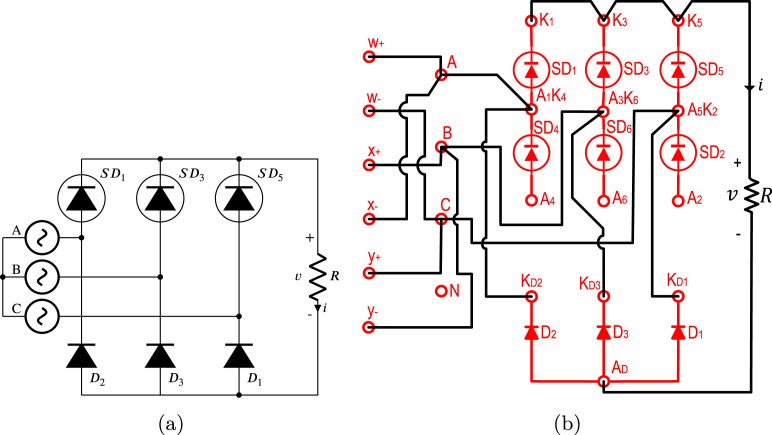
TPHWR with load R. (a) Simplified schematic diagram. (b) Connections on the front panel of the module.

**Fig. 21:**
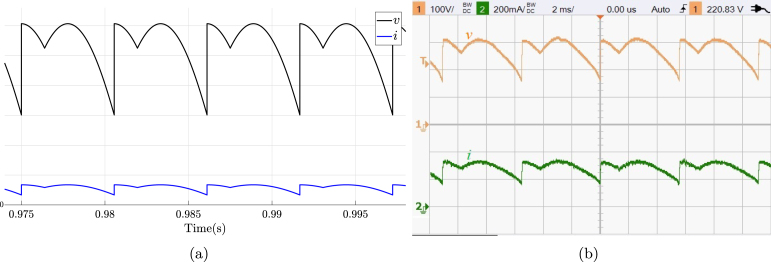
Waveforms v, and i for TPHWR with R load. (a) Simulation; v: 50 V/div, i: 0.5 A/div. (b) Experimental results.

TPHWR is a converter circuit AC–DC obtained from the TPFWR by replacing the $SD_{s}$ of the lower half by power diodes, as a result, the period of the TPHWR output voltage waveform is only one third of the period in the TPFWR; likewise, the inverter operation is not possible. The TPHWR is more cost-effective since diodes are significantly less costly than $SD_{s}$; besides, they do not require control electronics for the delay angle. On the other hand, the TPHWR is used in the same applications as the TPFWR, except when its operation as an inverter is required. This subsection displays the use of the proposed SCRs module in an electrical or electronic engineering laboratory activity, which is based on connecting a resistor to the output of a TPHWR as shown in Fig. 20(a). The respective connections necessary for its implementation on the SCR module are shown in Fig. 20(b). In this activity, the following parameters were used: RMS voltage of 125 V for the grid (A,B,C), a resistance R equal to 900 Ω, and α of $\frac{\pi }{6}$. The activity begins with energizing the module’s power supply, followed by setting the desired value of α; finally, the module connects to the power grid (A,B,C). Fig. 21 shows current waveforms i and the voltage v in the load, obtained both in simulation and experimentally in the SCRs module. The functionality of the module was validated by comparing the experimental waveforms with the simulated ones, the results show a significant agreement, displaying the effectiveness of the module in this experiment.

### 7.4 Fourth experiment: TPVC with resistive load in star configuration

**Fig. 22:**
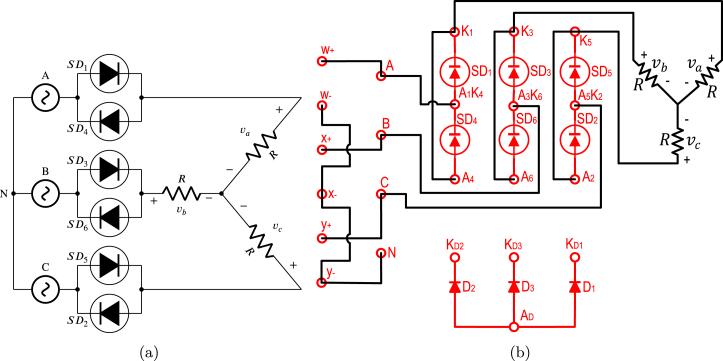
TPVC in star configuration. (a) simplified schematic diagram. (b) Connections on the front panel of the module.

**Fig. 23:**
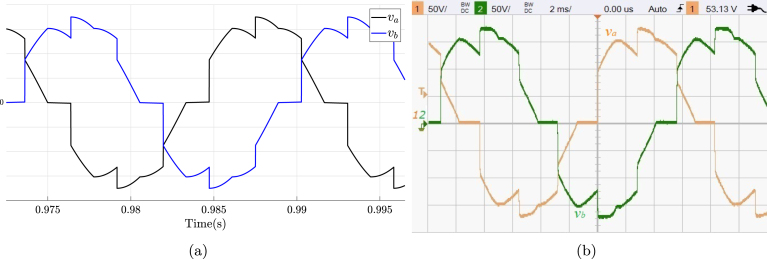
Waveforms $v_{a}$, and $v_{b}$ for TPVC in star configuration. (a) Simulation; $v_{a}$, and $v_{b}$: 50 V/div (b) Experimental results.

TPVC is an AC–AC converter circuit (also known as AC regulator) utilized to modify the RMS load voltage at a constant frequency achieving this adjustment through phase control and natural switching. Typical applications of TPVCs include the control of lighting and heating systems, tap changing in in-service transformers, as well as soft starting and speed regulation in pump and fan drives. This subsection shows the use of the proposed SCR module in an electrical or electronic engineering laboratory activity, which consists of connecting three resistors arranged in a star configuration to the output of a TPVC, as shown in Fig. 22(a). The corresponding connections required for its implementation on the SCR module are shown in Fig. 22(b). The parameters used in this activity are the following: RMS voltage of 125 V for the power grid (A,B,C), three resistors R in star configuration 480 Ω, and α equal to $\frac{\pi }{6}$. The activity begins with energizing the module power supply, followed by setting the desired value for α. Finally, the module is connected to the grid (A,B,C). Fig. 23 shows voltage waveforms $v_{a}$ and $v_{b}$ in the load obtained through simulation and experimentally. The functionality of the module was confirmed by contrasting the experimental waveforms with the simulated ones, displaying the agreement in the results. This consistency allows to verify the effectiveness of the module in this experiment.

### 7.5 Fifth experiment: RPSC monophase implementation

**Fig. 24:**
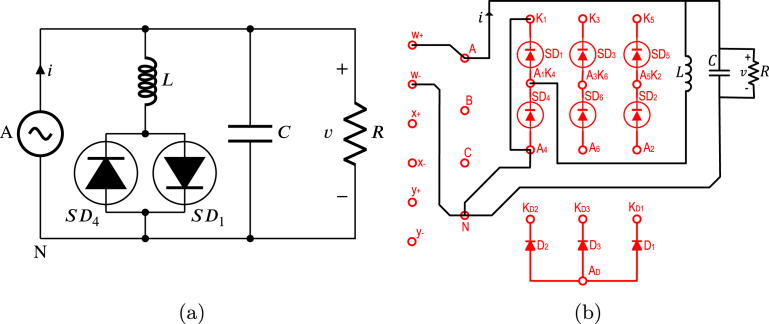
RPSC single-phase implementation. (a) simplified schematic diagram. (b) Connections on the front panel of the module.

Typically, capacitors are used in parallel with inductive loads to improve power factor. When the reactive power requirement for a load is constant, selecting a fixed-value capacitor to adjust the power factor to unity is feasible. However, when changes in reactive power occur, the choice of a fixed capacitor will result in a variation of the power factor. The circuit depicted in Fig. 24(a) represents the application of a single-phase AC–AC voltage controller that maintains the power factor close to unity, even when reactive power values fluctuate in the load. Here, the capacitor provides a constant reactive power, while depending on the delay angle the inductance absorbs a variable reactive power. The reactive power resulting from the interaction between the inductor and the capacitor is adjusted to reach a power factor close to unity. This subsection presents the application of the proposed SCRs module in an electrical or electronic engineering laboratory activity. This activity aims to vary the power factor through a single-phase AC–AC voltage controller. Fig. 24(b) shows the necessary connections for the implementation of this experiment on the SCRs module. In this activity, the parameters used were RMS voltage of 125 V for the line A, a resistance R of 90 Ω, a capacitor C equal to 15 µF and an inductance L of 0.46 H with an equivalent series resistance of 10 Ω due to copper loss. To carry out this activity; first, the module power supply is energized; second, α is set to π; third, the module is connected to line voltage A; Finally, the gradual decrease of α begins, obtaining the values detailed in Table 2. The operation of the SCR module was verified through a comparison between the experimentally measured power factor and the simulated result (displayed in Table 2); therefore, there is consistency between the experimental and simulated results, supporting the validity of the module.

**Table 2:** Power factor in the single-phase static reactive compensator.

PF (Experimental)	PF (Simulation)	α
0.868	0.896	π
0.868	0.896	$\frac{17\pi }{18}$
0.870	0.897	$\frac{8\pi }{9}$
0.878	0.905	$\frac{5\pi }{6}$
0.890	0.917	$\frac{7\pi }{9}$
0.906	0.933	$\frac{13\pi }{18}$
0.932	0.953	$\frac{2\pi }{3}$
0.960	0.974	$\frac{11\pi }{18}$
0.980	0.991	$\frac{5\pi }{9}$
0.992	0.999	$\frac{\pi }{2}$

## 8 Discussion

Since the SCR is an essential element in circuits for the conversion of electrical energy into alternating current, having the necessary elements for teaching the operation of SCRs is a fundamental aspect in universities where electronic, electrical, and related engineering programs are available. The design of these types of educational modules must consider ease of use and versatility in the implementation of different experiments.

The previous sections displayed different experiments that can be implemented for teaching purposes. This module can be also used in research to examine particular aspects of conversion circuits, such as operating times, waveforms, power calculations, harmonics, and efficiency, among others. Particularly, regarding the use of the module in research, DCST (Fig. 18) and RPSC (Fig. 24) circuits are used in alternating current microgrids and power factor correction systems, both relevant research topics currently.

## 9 Conclusions

This document presented an experimental module for education and research of SCRs in power electronics, where different experiments with a progressive level of complexity are proposed. It begins with experiments with a controlled and semi-controlled rectifier and ends with a more complex experiment for direct current power transmission with reactive power compensation using AC–AC voltage controllers. Applications with resistive load and active load are also considered.

For the TPFWR experiment with R−L load, the functionality of the module was checked by comparing the experimental waveforms with the simulated ones. The results show a significant agreement, displaying the module’s effectiveness in this experiment.

In a second experiment, a DCST was implemented, which is a system used in direct current transmission lines for power transmission over long distances. A notable coherence is observed between the experimental and simulated results, confirming the validity of the module in this experiment.

The third experiment consists of a TPHWR, which is an AC–DC converter circuit obtained from the TPFWR. The functionality of the module was validated by comparing the experimental waveforms with the simulated ones. The results show a significant agreement, demonstrating the module’s effectiveness in this experiment.

In the fourth experiment, a TPVC with a resistive load is arranged in a star configuration. This AC–AC converter circuit, also known as an AC regulator, is used to vary the RMS load voltage at constant frequency. In this case, the module’s functionality was confirmed by contrasting the experimental waveforms with the simulated ones, revealing a marked agreement in the results.

The single-phase RPSC experiment consists of a single-phase AC–AC voltage controller application that maintains a power factor close to unity, even when reactive power values vary across the load. The operation of the SCRs module was verified through a comparison between the experimentally measured power factor and the simulated power factor, showing a remarkable agreement between the experimental and simulated results, supporting the validity of the module in this experiment.

This work seeks to generate a reference framework for the development of experiments with SCRs in the field of teaching and research. The module is intended to be open source, allowing access to the DSP’s resources to implement the circuits and the programming software.

It is expected in future work to extend this type of modules to more general open source power electronics applications to contribute to the community interested in energy conversion research.

## CRediT authorship contribution statement

**Jhon Bayona:** Writing – review & editing, Writing – original draft, Validation, Software, Methodology, Investigation, Formal analysis, Conceptualization. **Nancy Gélvez:** Writing – review & editing, Writing – original draft, Methodology, Investigation, Conceptualization. **Helbert Espitia:** Writing – review & editing, Writing – original draft, Supervision, Methodology, Investigation, Conceptualization.

## Declaration of competing interest

The authors declare that they have no known competing financial interests or personal relationships that could have appeared to influence the work reported in this paper.
